# A small mitochondrial protein present in myzozoans is essential for malaria transmission

**DOI:** 10.1098/rsob.160034

**Published:** 2016-04-06

**Authors:** Dennis Klug, Gunnar R. Mair, Friedrich Frischknecht, Ross G. Douglas

**Affiliations:** Integrative Parasitology, Center for Infectious Diseases, Heidelberg University Medical School, Im Neuenheimer Feld 324, 69120 Heidelberg, Germany

**Keywords:** alveolates, apicomplexa, *Plasmodium*, transmission, mitochondrion, ookinete

## Abstract

Myzozoans (which include dinoflagellates, chromerids and apicomplexans) display notable divergence from their ciliate sister group, including a reduced mitochondrial genome and divergent metabolic processes. The factors contributing to these divergent processes are still poorly understood and could serve as potential drug targets in disease-causing protists. Here, we report the identification and characterization of a small mitochondrial protein from the rodent-infecting apicomplexan parasite *Plasmodium berghei* that is essential for development in its mosquito host. Parasites lacking the gene mitochondrial protein ookinete developmental defect (*mpodd*) showed malformed parasites that were unable to transmit to mosquitoes. Knockout parasites displayed reduced mitochondrial mass without affecting organelle integrity, indicating no role of the protein in mitochondrial biogenesis or morphology maintenance but a likely role in mitochondrial import or metabolism. Using genetic complementation experiments, we identified a previously unrecognized *Plasmodium falciparum* homologue that can rescue the *mpodd(−)* phenotype, thereby showing that the gene is functionally conserved. As far as can be detected, *mpodd* is found in myzozoans, has homologues in the phylum *Apicomplexa* and appears to have arisen in free-living dinoflagellates. This suggests that the MPODD protein has a conserved mitochondrial role that is important for myzozoans. While previous studies identified a number of essential proteins which are generally highly conserved evolutionarily, our study identifies, for the first time, a non-canonical protein fulfilling a crucial function in the mitochondrion during parasite transmission.

## 1. Background

The superphylum Alveolata consists of a large collection of eukaryotic organisms representing diverse living environments, morphologies and trophic strategies [1]. In many cases, these organisms have diverged from typically canonical cellular processes and arrangements in order to survive and reproduce in their corresponding niches. For instance, the mitochondria of apicomplexan parasites and related phototrophic chromerids such as *Vitrella* and *Chromera* are characterized by a reduced genome size when compared with ciliates such as *Tetrahymena* [2]. There is also a notable absence of some classical units of the electron transport chain whereas some enzymes, such as branched-chain ketoacid dehydrogenase, have been assigned to additional non-classical enzymatic roles (for recent reviews, see [3,4] as well as [5,6]). Thus, these organisms have evolved divergent approaches and factors to accomplish the necessary metabolism required for their biology and could provide unique targets for drug development in the case of disease-causing protists.

Malaria is caused by the obligate intracellular apicomplexan *Plasmodium*, a unicellular organism with a complex life cycle that requires a mosquito vector and a vertebrate host. To achieve transmission, the parasite possesses a genetic repertoire that enables it to switch between different host environments, for example the 37°C circulatory system of the mammalian host and the reduced temperature of the mosquito midgut. During the course of blood-stage infection, a subset of the parasite population develops into gametocytes—sexually immature forms under cell cycle arrest. Upon ingestion by the mosquito vector, the gametocytes rapidly mature into free gametes and male–female fusion results in zygote formation. Subsequently, zygotes develop into ookinetes: motile cells capable of breaching the peritrophic matrix surrounding the blood meal, traversing the mosquito midgut epithelium and converting into oocysts at the basal lamina. Within these oocysts, sporozoites develop that are transmitted back to the mammalian host. The different parasite forms exhibit strikingly different morphologies and express many stage-specific genes [7–10]. An array of factors contribute to the shape alteration of the zygote into the ookinete, including regulated kinase-dependent signalling [11–20], regulation of gene expression [21,22], localization of pellicular proteins required for ookinete shape [23,24] as well as metabolic activity of the parasite mitochondrion [25–30].

During the transmission from mammal to mosquito, the metabolic requirements for the parasite change and, as a result, there is an observable alteration of mitochondrial morphology and activity: in asexual blood stage mitochondria are acristate, and metabolism is assumed to be predominantly achieved by glycolysis, whereas in mosquito stage the mitochondria are well developed to perform tricarboxylate cycling and oxidative phosphorylation [27,31–34]. Accordingly, some genes required for the TCA cycle, electron transport chain and oxidative phosphorylation are not required for normal blood-stage development, but are nonetheless essential for the formation of infectious ookinetes and subsequent mosquito infection [25–30]. However, the full array of factors required for proper functioning of the *Plasmodium* mitochondrion still remains unknown.

Here, we report the discovery of a small mitochondrial transmembrane domain protein PBANKA_1222200 and its subsequent characterization in *Plasmodium*. The protein was named mitochondrial protein ookinete developmental defect (MPODD) owing to the localization of the protein and the observed phenotype when the gene is deleted: *mpodd(−)* parasites fail to infect the mosquito vector and display a defect in the developmental transition from zygotes to ookinetes in the rodent-infecting parasite *P. berghei*. Importantly, we identified the currently unannotated corresponding *P. falciparum* homologue implying that the role of this protein is also relevant to malaria parasites infecting humans. Using bioinformatics, we have identified homologues in many other apicomplexans as well as in the related chromerid *Vitrella brassicaformis* and the dinoflagellates *Perkinsus marinus, Oxyrrhis marina, Karlodinium veneficum* and *Alexandrium tamarense*, showing that this protein probably has a conserved mitochondrial role in myzozoans that already arose in non-parasitic alveolates.

## 2. Material and methods

### 2.1. Bioinformatic analysis

*Plasmodium* sequences were obtained from PlasmoDB (http://plasmodb.org/plasmo/, version 26) and multiple sequence alignments were done using Clustal Omega (http://www.ebi.ac.uk/Tools/msa/clustalo/). Potential signal peptides, secondary structures and transmembrane (TM) domains were predicted using SignalP (http://www.cbs.dtu.dk/services/SignalP/), TMHMM (http://www.cbs.dtu.dk/services/TMHMM-2.0/), TMPred (http://embnet.vital-it.ch/software/TMPRED_form.html), HHPred (http://toolkit.tuebingen.mpg.de/hhpred) and dense alignment surface DAS method [35]. The mitochondrial localization prediction algorithm MitoProt II (v. 1.101) was employed to assess possible export to this organelle [36]. Phylogenetic trees were created using iTOL (http://itol.embl.de/), and the molecular weight of probed proteins was calculated with Expasy (http://web.expasy.org/compute_pi/).

### 2.2. Statistical analysis

Statistical analysis was performed using GraphPad Prism v. 5.0 (GraphPad, San Diego, CA). Datasets with more than two conditions were tested with a one-way ANOVA. Normally distributed datasets limited to two groups were tested by using Student's *t*-test, whereas non-parametric sets were tested using a Mann–Whitney test. A value of *p* < 0.05 was considered significant.

### 2.3. Experimental animals and parasite lines

Female four- to six-week-old NMRI and C57Bl/6 mice were obtained from Janvier laboratories. For all transfections and genetic modifications, the *P. berghei* ANKA strain was used as recipient line [37].

### 2.4. Generation of parasite lines *mpodd(−)*, *mpodd::mCh*, *mpodd(−);mpodd^PBANKA^* and *mpodd(−);mpodd^PF3D7^*

*mpodd(−)* parasites were generated by amplifying 1132 bp upstream of PBANKA_1222200 via PCR with the primers 1 and 2 (electronic supplementary material, table S1). The PCR product was subcloned in the pGEM-T Easy vector (Promega) and mutated using site-directed mutagenesis (primers 7 and 8; electronic supplementary material, table S1) to remove a single restriction site for *Nde*I. This was required to clone the 5′ UTR of PBANKA_1222200 directly in front of mCherry to track promoter activity *in vivo*. The mutated 5′ UTR was cloned via *Sal*I and *Nde*I sites into the Pb262CSmChef1ahDHFRyFCU vector that contains the mCherry gene and the positive–negative selection marker *hdhfr-yfcu* [38]. To introduce the second site for homologous recombination, the fragment 632 bp downstream of PBANKA_1222200 was amplified using the primers 5 and 6 (electronic supplementary material, table S1) and cloned into the Pb262-PBANKA1222200KO-int vector via *Hind*III and *Xho*I. The final vector Pb262-PBANKA1222200KO was digested (*Sal*I and *Xho*I) and transfected into *P. berghei* ANKA parasites [39,40], giving rise to the *mpodd(−)* line. C-terminally mCherry-tagged parasites were generated in the same way with the exception that a different primer combination (primers 3 and 4; electronic supplementary material, table S1) was used to amplify the 5′UTR together with the open reading frame (ORF) of PBANKA_1222200. The final vector Pb262-PBANKA1222200TAG was transfected into *P. berghei* ANKA parasites and selected with pyrimethamine to generate the *mpodd::mCh* line. To complement knockout parasites, the PBANKA_1222200 ORF was amplified with flanking regions upstream and downstream using the primers 1 and 6 (electronic supplementary material, table S1). The PCR product was subcloned in the pGEM-T Easy vector and fully sequenced (GATC-Biotech Ltd, Konstanz) to ensure the sequence was free of mutations. The resulting pGEM-*Pb*Comp vector was digested (*Sal*I and *Xho*I) and transfected into *mpodd(−)* parasites to generate the *mpodd(−);mpodd^PBANKA^* line. We used the gene in/marker out approach based on selection with 5-fluorocytosine (1.5 mg ml^−1^) to select for parasites that have taken up the desired DNA construct [41]. The ORF of the *P. falciparum* homologue was synthesized (GeneArt, Invitrogen Ltd) without the intron and cloned via the restriction enzymes *Nde*I and *Hind*III in the Pb262-PBANKA1222200KO vector to replace the mCherry gene and the positive–negative selection marker *hdhfr-yfcu*. In a further step, the 3′ UTR of PBANKA_1222200 was amplified via PCR using the primers 6 and 9 (electronic supplementary material, table S1) and cloned into the Pb262-PF3D7Comp-int vector. The resulting Pb262-PF3D7Comp vector was digested (*Sal*I and *Xho*I) and transfected into the *mpodd(−)* line. Selection with 5-fluorocytosine gave rise to *mpodd(−);mpodd^PF3D7^* parasites. All used parasite lines in this study were cloned by limiting dilution to generate isogenic populations. Mice infected with parasites obtained from transfections (parental population) were grown to a parasitaemia of 0.3–1%. Donor mice were sacrificed, and blood was taken by cardiac puncture. Blood-stage parasites were diluted in PBS to 0.8 parasites per 100 µl. A total volume of 100 µl of the last dilution step was injected intravenously into 10 NMRI mice. Blood of infected mice was smeared from day 7 to day 14 [39]. Each mouse that was parasite-positive was assumed to be initially infected with a single parasite. Once parasitemia reached approximately 2%, blood was taken and parasites were either frozen for re-infections or purified to isolate genomic DNA. Genomic DNA was isolated using the Blood and Tissue Kit (Qiagen Ltd). Subsequently, obtained parasites were genotyped via PCR (electronic supplementary material, figure S1 and S2). Blood-stage growth rates were calculated during the course of limiting dilutions. Blood smears of infected mice were performed on day 8 post-injection of a single blood-stage parasite and the calculation for asexual growth rates determined using the general formula
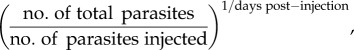
thus simplified to (no. of total parasites)^1/8^ [42].

### 2.5. Gene model confirmation of *Plasmodium berghei* and *Toxoplasma gondii mpodd*—identification of *Plasmodium falciparum mpodd*

Total RNA from mixed blood-stage *P. berghei* parasites and *P. falciparum* as well as *T. gondii* cultures was isolated with TRIzol reagent and reverse transcribed with Superscript II in the presence of oligo d(T) and random hexamers. cDNA was PCR-amplified with primers 18 and 19 for *P. berghei mpodd*, primers 24 and 25 for *P. falciparum mpodd*, and primers 22 and 23 for *T. gondii mpodd* (electronic supplementary material, table S1). Amplicons were subcloned into pGEM (*P. berghei* and *P. falciparum mpodd*) or L432 and sequenced to confirm the identity of the transcribed product.

### 2.6. Life cycle stage-specific reverse transcriptase-PCR

Total DNA and RNA of parasites at different life cycle stages were isolated using the TRIzol reagent according to the manufacturer's protocol (Thermo Scientific). cDNA was prepared from isolated RNA using the Superscript II kit. Primers used for PCR amplification of the *mpodd* locus using Taq polymerase are given in electronic supplementary material, table S1.

### 2.7. Western blot

To verify expression of the MPODD::mCherry fusion protein, saponin pellets of schizont cultures from *mpodd::mCh* and *mpodd(−)* parasites were lysed in 50 µl RIPA buffer (50 mM Tris pH 8, 1% NP40, 0,5% sodium dexoycholate, 0,1% SDS, 150 mM NaCl, 2 mM EDTA). Probes were mixed and denaturated with Laemmli buffer and separated on 4–15% SDS–PAGE gels (Mini Protein TGX Gels, Bio-Rad). Gels were blotted on nitrocellulose membranes using the Trans-Blot Turbo Transfer System (Bio-Rad) and incubated over night with antibodies directed against mCherry (rabbit polyclonal antibody, 1 : 5000 dilution, ab183628 from AbCam) or HSP70 (mouse monoclonal antibody, 1 : 5000 dilution, obtained through the MR4 as part of the BEI Resources Repository, NIAID, NIH: Mus musculus 65, MRA-662, MF Wiser). Secondary anti-rabbit (Immun-Star (GAR)-HRP, Bio-Rad) and anti-mouse antibodies (NXA931, GE Healthcare) conjugated to horseradish peroxidase were applied subsequently for 1 h (1 : 10 000 dilution). Signals were detected using SuperSignal West Pico Chemiluminescent Substrate (Thermo Fisher Scientific).

### 2.8. Ookinete cultures

Mice were infected by intraperitoneal injection of frozen stocks and parasites allowed to multiply to a parasitaemia of 1.2–2%. Twenty million parasites were transferred to two naive mice. Three days after transfer, efficient transmission ability was assessed by observation of exflagellation events. One drop of infected mouse blood was placed on a microscope slide for 10 minutes at 20°C. Exflagellation events were counted using a light microscope (Carl Zeiss GmBH, Jena, Germany) with a counting grid. If at least two events per field of view (under 40× magnification) were observed, mice were bled by cardial puncture, blood transferred to T-75 cell culture flasks (NUNC Corp) and mixed with 12 ml complete ookinete medium (RPMI-1640, 25 mM Hepes, 300 mg l^−1^, l-glutamine, 10 mg l^−1^ hypoxanthine, 50 000 units l^−1^ penicillin, 50 mg l^−1^ streptomycin, 2 g l^−1^ NaHCO_3_, 20.48 mg l^−1^ xanthurenic acid, 20% foetal bovine serum, pH 7.8). Cultures were incubated for 20 h at 19°C. For localization studies, ookinetes were used directly from culture. For morphology observations, cultures were transferred to 15 ml tubes and centrifuged for 8 min at 1500 rpm at room temperature. Supernatants were discarded, and red blood cells lysed by the addition of 10 ml of ice-cold 170 mM NH_4_Cl. After incubation of 10 min on ice, ookinetes were pelleted by 8 min of centrifugation at 1500 r.p.m. (4°C). Pellets were washed with 10 ml HBSS (GE Healthcare Life Sciences) and centrifuged again as above. Supernatants were discarded, and the remaining pellets were resuspended in 1 ml ookinete medium. The suspensions were either smeared and stained with Giemsa solution to test for developed ookinetes or directly imaged using a Axiovert 200M (Carl Zeiss GmBH, Jena, Germany) fluorescence microscope.

### 2.9. Electron microscopy

Ookinetes were produced as outlined above and purified from uninfected erythrocytes by gradient purification centrifugation on a 63% Nycodenz (in PBS) cushion. In order to more easily visualize the mitochondrial network, we adapted a 3,3′-diaminobenzidine (DAB) staining procedure [43,44]. Cells were fixed in 4% glutaraldehyde (0.1 M phosphate buffer pH 7.2) for approximately 1 h at 4°C. Fixed cells were then washed extensively (twice washed with 0.1 M phosphate buffer pH 7.2, followed by twice washed with 0.1 M Tris buffer pH 7.5) and resuspended in a solution containing 2 mg DAB and 0.06% (v/v) H_2_O_2_ in 0.1 M Tris buffer pH 7.5. The reaction was allowed to proceed at room temperature (in the dark) for approximately 80 min and was then washed once with an equivalent volume of distilled water. Prepared cells were postfixed with 1.5% KMnO_4_ for 20 min at room temperature, rinsed in water and stained overnight in 0.5% uranyl acetate at 4°C. In preparation for imaging, samples were serially dehydrated with ethanol and embedded in Epon (60°C for 8 h) followed by sectioning. Images were acquired on a JEOL JEM-1400 electron microscope at 80 kV using the TemCam F416 (Tietz Video and Image Processing Systems GmBH, Gautig).

### 2.10. Mosquito infections and analysis

Two donor mice were infected by intraperitoneal injection of frozen stocks. Transmission potential was estimated by observing exflagellation events as described above. When at least one exflagellation event per counting field was observed, mice were anaesthetized and fed to mosquitoes for 30 min. Prior to feeding, mosquitoes were starved overnight to ensure maximal bite rates. Infected mosquitoes were kept at 80% humidity and 21°C in a climate chamber until analysed.

To observe the development of oocysts, midguts of 20–30 mosquitoes were isolated at day 12 post-infection. Midguts were dissected in PBS and either directly mounted on a microscope slide for live cell imaging or stained with 0.1% mercurochrome solution for 30 min followed by washing of the stained midguts with PBS [45]. The number of oocysts within stained midguts was counted manually with 10-fold magnification using an Axiovert 200M fluorescence microscope. To investigate the sporozoite stage, salivary glands of infected mosquitos were isolated between day 17 and day 24. Salivary glands were dissected in RPMI (supplemented with 50 000 units l^−1^ penicillin, 50 mg l^−1^ streptomycin), crushed to release sporozoites and purified with 17% Accudenz as described previously [46]. Purified sporozoites were resuspended in RPMI further supplemented with 3% bovine serum albumin (ROTH Ltd) and transferred into a 96-well plate.

### 2.11. Protein localization

A mCherry-tagged MPODD construct (electronic supplementary material, figure S1) was used for the assessment of protein localization. For all parasite life stages, approximately 200 nM MitoTracker Green FM (Life Technologies, *λ*_ex_ 490 nm, *λ*_em_ 516 nm) was employed in line with the manufacturer's recommendations. For blood-stage parasites, a drop of blood from an infected mouse was added to a microscope slide together with the addition of MitoTracker and 1 µg ml^−1^ Hoechst 33342 DNA dye. Two hundred microlitres of cultured ookinetes were centrifuged for 10 s at 13 000 r.p.m. (room temperature) and resuspended in 20 µl culture medium containing MitoTracker and Hoechst at the above-mentioned concentrations. Purified salivary gland sporozoites were resuspended in 100 µl RPMI supplemented with 3% BSA and the above-mentioned dyes. Two hundred nanomolar cytochalasin D was also included in the sporozoite medium to minimize motility of activated parasites. For liver stage imaging, approximately 20 000 freshly isolated sporozoites were added to Huh7 cells at approximately 70% confluency for 30 min. The cells were then washed and allowed to culture for 28 h at 37°C, 80% relative humidity and 5% CO_2_. Cells were then incubated in MitoTracker and Hoechst for 30 min at the above-mentioned concentrations and then replaced with regular culture medium (DMEM, 10% FBS and 2 mM glutamine). All images were acquired using a spinning disc confocal microscope (Nikon Ti series) with 100-fold magnification (NA 1.4). In order to assess whether the mitochondria of knockout ookinetes had any residual activity as detected by the dye, ookinete cultures of both the knockout and wild-type strains were produced and stained as described above. Approximately 50 ookinetes from each culture were imaged under the same conditions and durations, and the mitochondrial staining measured using the ImageJ ‘measure’ plugin in FIJI [47].

### 2.12. Estimation of prepatencies by native transmission and sporozoite injections

To assess any possible defects during transmission from vector to host, re-infection experiments were performed. Infected mosquitos between day 17 and day 24 post-infection were separated into cups to 10 each and starved for 6–8 h. Per experiment, four four- to six-week-old C57Bl/6 mice were anaesthesized using 70 µl of a mixture between ketamine and xylazine (87.5 mg kg^−1^ ketamine, 12.5 mg kg^−1^ xylazine). One anaesthetized mouse was put on each cup, and mosquitoes were allowed to bite for 10 min on the ventral and dorsal side. Twenty-four hours after feeding, mosquitos that had taken a blood meal were dissected to determine sporozoite numbers within salivary glands and midguts. Additionally, salivary gland sporozoites were dissected from mosquitos at day 17 to day 24 post-infection. Infected salivary glands were stored in RPMI medium (containing 50 000 units l^−1^ penicillin, 50 mg l^−1^ streptomycin) and crushed with a pestle to release the sporozoites. The number of sporozoites was counted (in a Neubauer counting chamber) and subsequently diluted to 10 000 sporozoites per 100 µl. For each experiment, four C57Bl/6 mice were injected intravenously with 10 000 sporozoites. The blood of bitten and injected mice was smeared from day 3 to day 10 after injection or exposure to mosquitoes. Taken smears were stained in Giemsa solution (Merck KGaA) and counted using a light microscope with a counting grid. The time between infection and observation of the first parasite within a blood smear was calculated as prepatency.

## 3. Results

### 3.1. Mitochondrial protein ookinete developmental defect (PBANKA_1222200) is a predicted transmembrane protein that is present throughout the *Plasmodium* life cycle and localizes to the mitochondrion

**Figure 1.**
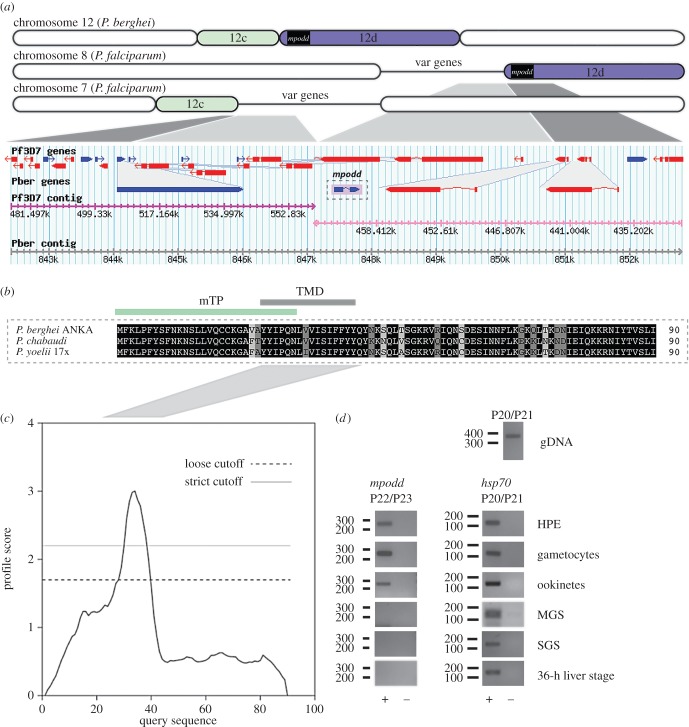
*mpodd* codes for a transmembrane protein and is located on a synteny break between rodent and human malaria species. (*a*) Organization of *P. berghei* chromosome 12 in comparison to chromosomes 7 and 8 from *P. falciparum* strain *3D7* (not drawn to scale)*.* Evolutionary chromosome rearrangements have resulted in fragmentation of chromosome 12. Therefore, the equivalent segment of *P. berghei* 12d localizes to chromosome 8 in *P. falciparum* while segment 12c localizes on chromosome 7. As indicated, *mpodd* is the first protein coding gene on segment 12d. Sites of crossing-over events are marked by congregations of *var* genes. Gene locus of *mpodd* retrieved from PlasmoDB is shown below the scheme: genes are indicated as blue and red arrows, breaks within arrows indicate introns. Corresponding contigs are indicated below the genes. (*b*) Multiple sequence alignment of MPODD from *P. berghei ANKA* with its homologues in *P. chabaudi*, and *P. yoelii* 17X. Conserved residues are written in white and highlighted in black, highly conserved residues are highlighted in dark grey and mostly conserved residues are highlighted in light grey. Predictions (§2.1) indicate the presence of a transmembrane domain (TMD) and a mitochondrial targeting peptide (mTP) shown as green and black line above the alignment. The alignment was performed with the multiple sequence alignment tool Clustal Omega (http://www.ebi.ac.uk/Tools/msa/clustalo/). (*c*) Hydrophobicity plot (dense alignment surface method) based on the sequence of *Pb* MPODD indicates the presence of a transmembrane domain between tyrosine 25 and tyrosine 40. (*d*) Total RNA was isolated from indicated life cycle and used in RT-PCR with *mpodd*-specific primers; (+) and (−) indicate RT+ and RT− cDNA synthesis reactions. Primers specific for *hsp70* were used as an expression control for all stages. Amplification of genomic DNA (gDNA) shows the presence of an intron. HPE, non-gametocyte producer line/asexual stage parasites. MGS, midgut sporozoites. SGS, salivary gland sporozoites.

We and others routinely integrate Pb238/262 expression vectors into a presumed silent intergenic *P. berghei* region. This vector targets a region on chromosome 12 (position 820 043–821 271 bp; PlasmoDB version 26) that represents a synteny break between rodent malaria parasites and the human malaria parasite *P. falciparum* [48,49] (figure 1*a*). During the generation of a set of transgenic *P. berghei* parasites, we noted a marked decrease in transmission efficiency following removal of the resistance marker by negative selection (electronic supplementary material, figure S3). We hypothesized that the bidirectional nature of the *ef1α* promoter maintains sufficient transcription of a neighbouring, yet at the time unknown gene. Indeed, a recent updated annotation of the *P. berghei* genome indicated the presence of a previously unrecognized gene (PBANKA_1222200, chromosome 12, position 820 970–821 361 kb) immediately downstream of the integration site. We therefore characterized this gene and experimentally determined its role for life cycle progression. The gene consists of one intron and two exons encoding for a 90-amino-acid protein. Syntenic homologues have been identified in the two related rodent malaria species *P. yoelii* and *P. chabaudi* (figure 1*b*) but are notably absent from *P. vivax*, *P. knowlesi* and *P. falciparum*, and the gene is thus currently annotated as conserved rodent malaria protein with unknown function. The gene is located at a synteny break point between rodent malaria species and human infecting *P. falciparum*. The *P. falciparum* locus is characterized by multiple *var* gene copies: protein encoding genes important for antigenic variation [48]. Owing to the phenotype of the deletion mutants and the localization of the protein (see below), we named the protein MPODD.

**Figure 2.**
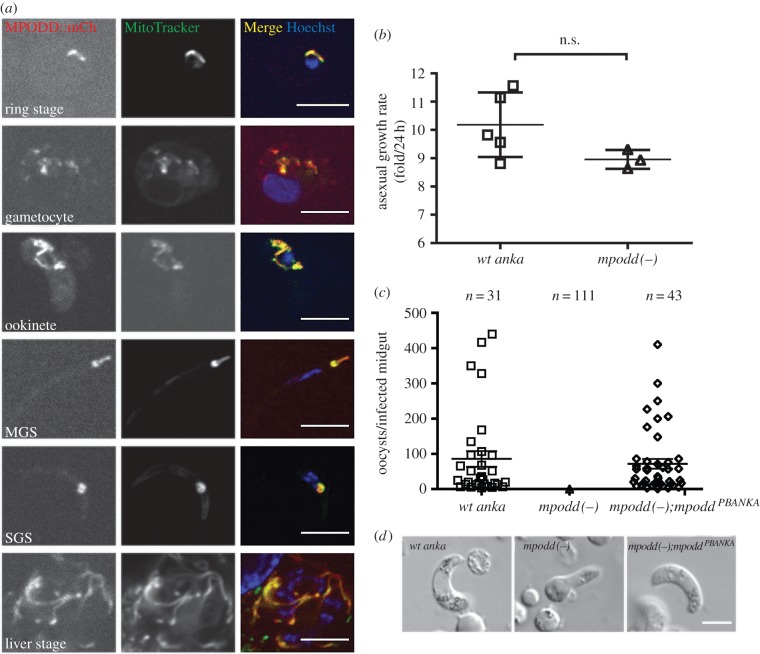
MPODD is localized to the parasite mitochondrion and is important for zygote to ookinete conversion. (*a*) C-terminally mCherry tagging of MPODD reveals expression in all life cycle stages and co-localization with MitoTracker Green-FM. MGS, midgut sporozoite. SGS, salivary gland sporozoite. (*b*) *mpodd(−)* parasites display comparable blood-stage growth rates. (*c*) *mpodd(−)* parasites do not form oocysts in the mosquito midgut, whereas complementation of the knockout with *Pb* MPODD (*mpodd(−);mpodd^PBANKA^*) restores infectivity. Mean with standard error indicated by the horizontal lines. The average *wt anka* oocyst load per infected midgut was not significantly different from *mpodd(−);mpodd^PBANKA^* infected mosquitoes. (*d*) *mpodd(−)* parasites do not develop into fully mature ookinetes and arrest at the retort stage. Scale bar: 5 µm.

Bioinformatic analysis of MPODD revealed the presence of a TM domain as well as a mitochondrial targeting signal (figure 1*b*,*c*). In order to understand the role of MPODD throughout the parasite life cycle, we performed RT-PCR to confirm the gene model and determine its transcription profile. In addition, we tagged the endogenous MPODD C-terminally with mCherry (*mpodd::mCh*) to assess its localization throughout the parasite life cycle. Expression of the MPODD::mCherry fusion protein was verified by Western blot (electronic supplementary material, figure S4). The *mpodd* transcript was detected in blood stages and ookinetes (figure 1*d*), whereas the tagged protein was observed in all life stages with an apparent increased fluorescent signal in mosquito-stage parasites. MPODD::mCh localized to the mitochondrion at all life stages as seen by the distinct co-localization of the fusion protein with the dye MitoTracker (figure 2*a*).

### 3.2. *mpodd(−)* parasites display normal mammalian blood-stage growth, but show a developmental halt in the mosquito

In order to define the potential role for *mpodd* during life cycle progression, we generated a gene deletion mutant by replacing the entire ORF of *mpodd* with a pyrimethamine selection cassette following established methodologies (electronic supplementary material, figure S1). Gene removal did not affect parasite viability in the blood stages and asexual parasites grew at similar rates to the wild-type control (figure 2*b*), consistent with reduced mitochondrial functions during asexual development of the parasite in the blood stream. However, *mpodd(−)* parasites displayed a clear developmental defect when transmitted to the mosquito vector. Even though *mpodd(−)* parasites showed normal exflagellation of male gametes, independent mosquito feeding experiments did not result in the establishment of oocysts, revealing a complete block in parasite transmission in the gene deletion mutant (figure 2*c*) that could be due to defects in fertilization or ookinete development. Using standard ookinete *in vitro* cultures, we could show that knockout parasites failed to develop mature, infectious ookinetes. *mpodd(−)* mutants typically resembled retort forms with the nucleus still positioned in the bulb-like extension of the zygote (figure 2*d*), suggesting an important role in final conversion from zygotes to ookinetes. Complementation of the *mpodd(−)* line with the endogenous *P. berghei* gene rescued the phenotype completely, reconstituting the ability to produce infectious ookinetes and to produce oocysts in similar numbers per infected midgut to wild-type controls (figure 2*c*,*d*; electronic supplementary material, table S2).

### 3.3. Absence of MPODD does not affect mitochondrial integrity but results in reduced mitochondrial mass

**Figure 3.**
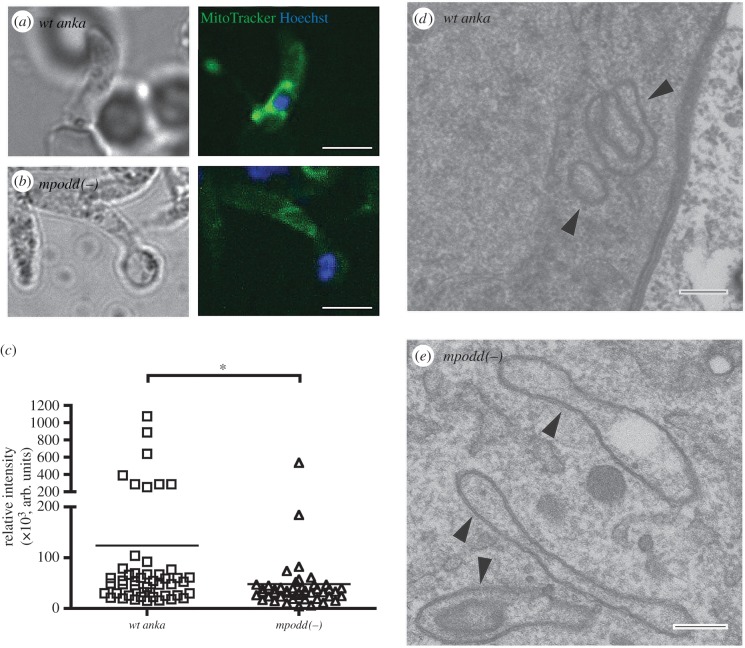
*mpodd(−)* ookinete mitochondria display reduced staining with MitoTracker Green FM dye, suggesting reduced mitochondrial mass. (*a*,*b*) Representative images of wild-type and *mpodd(−)*, respectively, stained with Hoechst and MitoTracker Green FM dye, showing diminished MitoTracker signal in *mpodd(−)* mitochondria compared with wild-type. Scale bar: 5 µm. (*c*) Quantification of fluorescence intensities of acquired pixels from a set area in the mitochondrial region were measured using the ‘measure’ plugin of FIJI. Individual points on the graph represent the average intensity of a single stained ookinete mitochondrion and the mean represented as a horizontal line. *p < 0.01 (Mann–Whitney test). (*d,e*) Representative images of glutaraldehyde fixed ookinetes subsequently stained with diaminobenzoic acid revealing comparable structural integrity between parasite lines. Arrowheads indicate stained mitochondrion regions. Scale bar for EM images: 200 nm.

Given the localization of MPODD to the mitochondrion, we hypothesized that MPODD could play an active role in this parasite organelle (either metabolically or structurally) once in the mosquito. To investigate this, we stained both wild-type and *mpodd(−)* ookinetes with MitoTracker Green FM dye [50]. This dye fluoresces selectively in the membrane of mitochondria in a membrane potential independent manner and shows the extent of mitochondrial mass in a cell [51,52]. Notably, the signal intensities of mitochondria staining in the knockout line were lower than the wild-type control suggesting reduced mitochondrial mass in the developing ookinete when the gene is absent (figure 3*a*–*c*). In order to assess the effect of *mpodd ­*deletion on mitochondrial integrity, we isolated ookinetes (or the underdeveloped forms in the case of the knockout), fixed them using standard methodologies and stained for mitochondrial peroxidase activity using DAB staining (see Materials and methods). Electron micrographs show comparable mitochondrial morphology in both the wild-type and knockout lines, suggesting that the absence of MPODD does not affect the overall structural integrity of the organelle as a whole (figure 3*d*,*e*).

### 3.4. Identification of a fully functional *Plasmodium falciparum* homologue and evolutionary origin of MPODD in alveolata

**Figure 4.**
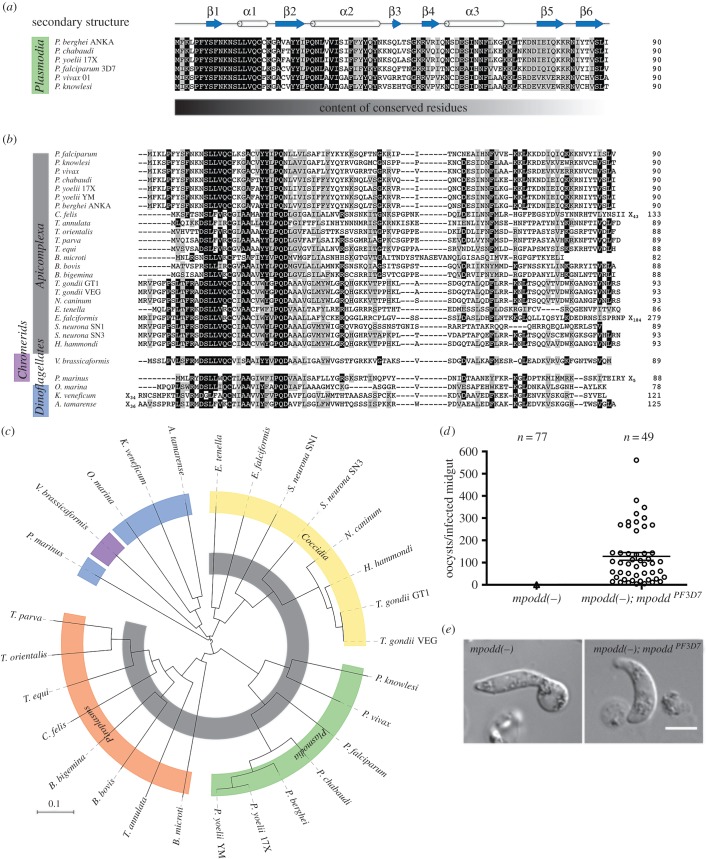
MPODD arose in a myzozoan ancestor and is functionally conserved. (*a*) Multiple sequence alignment of MPODD homologues from different *Plasmodium* species. The secondary structure predicted with HHPred is indicated above the alignment (arrows indicate beta-strands, whereas barrels indicate alpha-helices). Residues that are conserved are written in white and highlighted in black, mostly conserved residues are highlighted in grey. The sequences are especially conserved at the N-terminus, whereas the degree of conserved residues decreases towards the C-terminus (indicated below the alignment). (*b*) Alignment of all MPODD homologues identified within apicomplexans, chromerids and dinoflagellates. Phyla are indicated on the left. (*c*) Phylogenetic tree of the sequences aligned in (*b*). Colours for different subclasses and phyla correspond to (*a*) and (*b*), whereas *Piroplasmida* (orange) and *Coccidia* (yellow) are also indicated. (*d*) Oocyst numbers 12 days post-infection of *mpodd(−);mpodd^PF3D7^* in comparison with *mpodd(−)* infected mosquitoes. Mean with standard error indicated by the horizontal lines. (*e*) *mpodd(−);mpodd^PF3D7^* and *mpodd(−)* ookinetes after culturing for 20 h. The *mpodd* homologue from *P. falciparum* fully restores normal ookinete development.

MPODD is currently annotated as a conserved rodent malaria protein with unknown function in *P. berghei*, *P. chabaudi* and *P. yoelii*, and there are no predicted homologues in any of the human malaria parasites. Its key role in the mitochondrion of *P. berghei* for parasite transmission suggested that this important function might be evolutionarily conserved as the life cycle progression in rodent and human malaria parasites is similar, capable of infecting the same mosquito host. We therefore extended our search for MPODD homologues to other apicomplexan parasites. Using BLASTP, we identified a number of already annotated putative homologues at eupath.db with sequence similarities to *Pb* MPODD especially within the N-terminal half. Searching EST databases at NCBI with TBLASTN, we identified additional transcripts with coding potential for MPODD homologues in a number of apicomplexan parasites (with the exception of *Cryptosporidium* which lacks mitochondria and only has mitosomes) as well as phototrophic ancestors in the chromerid and dinoflagellate clades (figure 4*a*,*b*).

Importantly, upon re-visiting the genomic locus in *P. falciparum* and using TBLASTN, we identified a possible, unannotated *P. falciparum* homologue on chromosome 8; 425 171–424 797 bp (3D7 strain). The putative *P. falciparum* homologue *Pf* MPODD shares 62% identity to *P. berghei*. In this locus, the *P. falciparum* homologue is flanked by the same neighbouring genes as PBANKA_1222200 on chromosome 12. Transcription of the gene was supported by a number of ESTs, and we confirmed the conserved exon–intron organization by RT-PCR using cDNA prepared from cultured parasites. In addition, the gene organization of a homologue in *Toxoplasma gondii* without assigned function (chromosome X, 3690054–3691863 bp, GT1 strain, ToxoDB version 26) was confirmed by RT-PCR suggesting the encoding of the predicted protein (electronic supplementary material, S1).

Within the genus *Plasmodium*, the sequences adhere to a defined predicted secondary structure (figure 4*a*). A multiple sequence alignment identified a conserved N-terminal region across all detected myzozoan members with reduced sequence similarity across the C-terminus of the proteins, and a general length of approximately 90 amino acids (figure 4*b*). With the exception of *P. marinus*, a phylogentic analysis of MPODD recapitulates the established phylogenetic positions identified in a recent report [53] (figure 4*c*).

Finally, we introduced the newly identified *P. falciparum* homologue into the *mpodd(−)* parasite. Complementation with the proposed *P. falciparum* homologue from the 3D7 strain (termed *Pf mpodd*) completely rescued the phenotype of the knockout *P. berghei* line described above. *mpodd(−);mpodd^PF3D7^* parasites displayed fully formed ookinetes, and the ability to form oocysts in the mosquito vector was equivalent to wild-type and the *P. berghei* complemented line *mpodd(−);mpodd^PBANKA^* (figures 2*c* and 4*d*,*e*; electronic supplementary material, table S2).

## 4. Discussion

Myzozoans display significant divergence from their ciliate relatives. This is especially obvious in the case of the mitochondrion whereby the myzozoan genomes are markedly reduced with no more than three protein coding genes, the smallest identified [3,54]. Further, key complexes are often absent in these organisms, suggesting modified strategies for effective energy production. Here, we have identified MPODD, a small mitochondrial protein, as a key molecular player in the development of *Plasmodium* ookinetes and in mosquito-stage establishment. We developed an interest in this gene during the generation of a fluorescent reporter line, where we made use of available vectors that integrate into a region of chromosome 12 previously thought to be transcriptionally silent (position 820 043–821 271 bp) [49]. These transgenic lines behave normally throughout the life cycle [55,56]; however, upon removal of the resistance marker by negative selection [41], we noted a marked decrease in transmission to the mosquito, presumably by affecting *mpodd* expression. Importantly, while the gene is referred to as an uncharacterized ‘conserved rodent *Plasmodium* gene’, we have shown here that we can rescue the phenotype by complementation with a homologous gene that we have identified in *P. falciparum*. This suggests that there could be additional genes currently annotated as rodent malaria specific that are conserved across species, especially given that the numbers of each of this category varies between the annotated genomes (36 genes in *P. yoelli*, 21 genes in *P. chabaudi* and 15 genes in *P. berghei*). Indeed, a bioinformatic analysis of the 15 rodent-specific genes in *P. berghei* revealed that at least two of these genes (PBANKA_0406600 and PBANKA_1003900) probably have homologues in human infecting *Plasmodium* species. In addition, through bioinformatics of available sequences, we identified homologues of *mpodd* in *P. vivax* and *P. knowlesi* and transcription of a *T. gondii mpodd* homologue was confirmed by RT-PCR. A bioinformatic analysis of MPODD within the Alveolata resulted in the identification of numerous homologues in apicomplexans, dinoflagellates and the apicomplexan ancestral organism *Vitrella brassicaformis* (electronic supplementary material, figure S5) [53]. The phylogenetic distribution of MPODD among myzozoans suggests that the protein fulfils an ancient function within the mitochondrion of this clade. Interestingly, no homologues were identified outside of this clade including its sister group. While we cannot exclude the existence of *mpodd*-like homologues of lower sequence similarity elsewhere, our current analysis of available databases suggests that *mpodd* is only present in myzozoans.

MPODD possesses a mitochondrial targeting peptide and a putative TM region (figure 1*b*,*c*) suggesting transport into the mitochondria and localization of MPODD to the inner mitochondrial membrane. Interestingly, while the C-terminus is generally divergent in sequence, the N-terminal portion of the protein is well conserved between dinoflagellates, chromerids and apicomplexans. This suggests an important contribution of this region, either in mitochondrial targeting alone or together with its primary function.

MPODD displayed a distinct localization in the mitochondrion throughout the life cycle, indicating a specific function in this organelle. *mpodd* deletion had no observable effects in the blood stage, which is consistent with other studies assessing important mitochondrial proteins. Parasite mitochondrial activity is only residual in blood-stage parasites, with several knockout lines of mitochondrial enzymes displaying no significant phenotypic defect in blood-stage growth. Phenotypic effects are typically observed in early mosquito stages, largely in ookinete or oocyst formation [25–30]. This suggests a number of important factors are required for transitioning, in both metabolism and morphology, of the parasite mitochondrion during parasite transmission from mammal to arthropod. While these previous studies identified a number of essential proteins that are evolutionarily conserved, our study identifies for the first time, to the best of our knowledge, a non-canonical protein fulfilling a crucial function in the mitochondrion during parasite transmission.

The exact function of MPODD in this organelle is not fully understood. Interestingly, MitoTracker Green FM stains the mitochondria of knockout parasites less intensely than that of wild-type parasites. This suggests that there is significantly reduced mitochondrial mass in parasites lacking this protein. Our electron microscopy studies suggest that the overall integrity of the mitochondrion is comparable to controls. Alteration of organelle mass could be due to an indirect effect of altered metabolism in the cell. Indeed, it has been proposed that human T-effector cells that are more dependent on glycolysis (and less dependent on mitochondrial function) tend to have reduced mitochondrial mass [57]. We could therefore be observing the knockout line displaying less effective stage-specific mitochondrial respiration. Alternatively, reduced MitoTracker Green FM staining when *mpodd* is absent could indicate decreased protein levels in the mitochondrial membrane [52], and therefore an altered protein import profile, suggesting that MPODD could play a role in transport. MPODD homologues were identified in a wide variety of apicomplexans, chromerids and dinoflagellates, but its apparent absence in the apicomplexan *Cryptosporidium* is an important observation: this genus possesses a highly reduced mitochondrion and thus is predicted to have only minimal metabolic function [6,58]. *mpodd*'s absence there, together with our results, suggests that MPODD is not involved in the fundamental processes of mitochondrial biogenesis or iron-sulfur cluster formation, which are the remaining ‘mitochondrial’ functions assumed to be intact in *Cryptosporidium* [58]. We propose that, given the small size of the protein, MPODD possibly acts as an important scaffolding subunit within a larger protein complex that is presumably involved in metabolic function or transport of important metabolic factors (either proteins, lipids or ions) [5,59,60]. Identification of specific interaction partners (for example, using BioID methods) would clarify this mitochondrial role that assists us in understanding the divergent features of an array of organisms.

## Supplementary Material

Supplementary Figure S1 to S5

## Data accessibility

Bioinformatic details of all MPODD homologues used in this study, both for sequence comparisons and complementation experiments, can be found in the electronic supplementary material.
