# Supplementary material for: A small mitochondrial protein present in myzozoans is essential for malaria transmission
Source: Open Biol. 2016 Apr 6;6(4):160034. doi: 10.1098/rsob.160034 (PMC4852462; doi:10.1098/rsob.160034)
Supplement: Supplementary Figure S1 to S5 [file rsob160034supp1.pdf]

Supplementary information

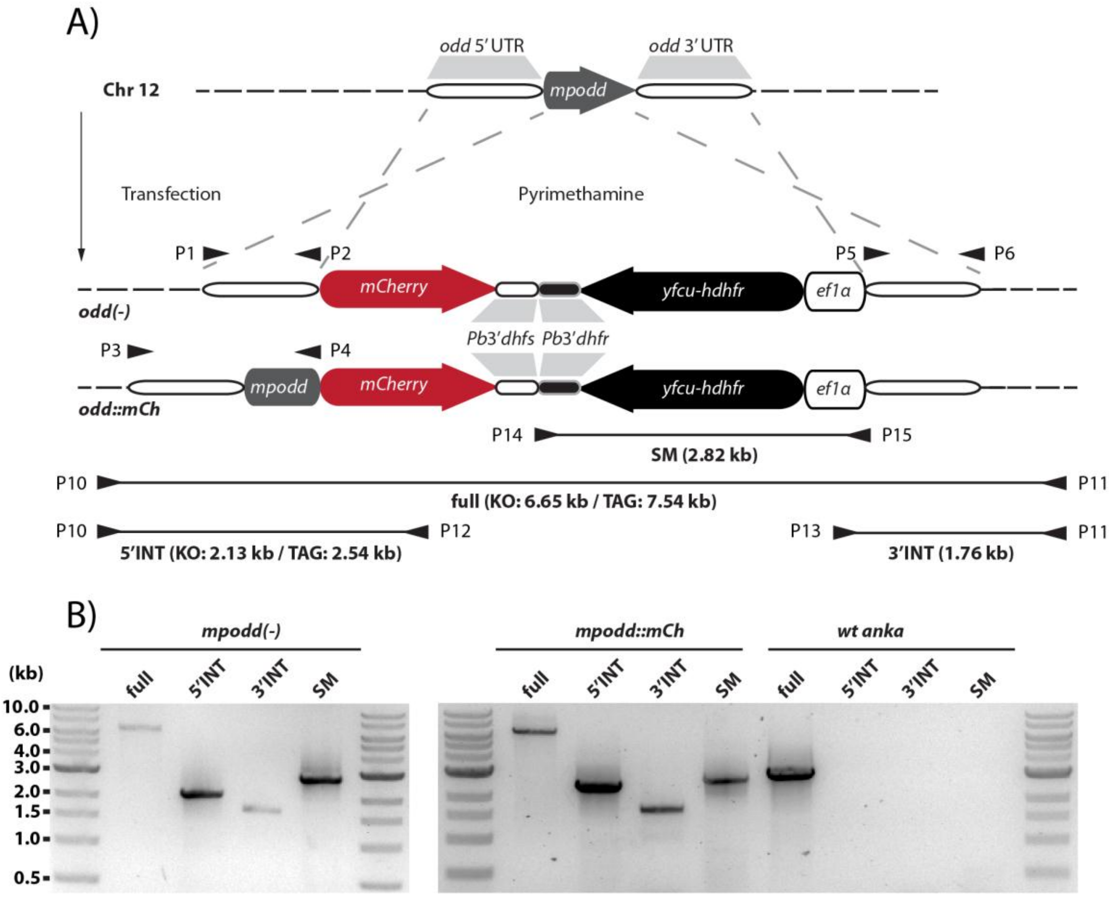

Figure S1. Generation and PCR analysis of *mpodd(-)* and *mpodd::mCh* parasite lines

Schematic representation of knockout and tagging strategy. A) *mpodd(-)* parasites were generated by transfection of wild-type (WT) ANKA with a vector containing the mCherry gene and a fusion of the positive selectable marker *hdhfr* (human dihydrofolate reductase) with the negative marker *yfcu* (yeast cytosine deaminase and uridyl phosphoribosyl transferase) under control of the *P. berghei ef1α* promoter and the *3'dhfr* terminator. This construct was flanked by 1kb sequences upstream and downstream of the *mpodd* gene to generate a replacement by double crossover homologous recombination. *mpodd::mCh* parasites were generated in the same way but the transfected DNA contained beside the 5' flanking sequence the complete open reading frame (ORF) of *mpodd* fused c-terminally to the mCherry gene. Parasites that successfully integrated the constructs were positively selected with pyrimethamine and cloned as previously described. Location of primers and

length of PCR fragments used for genotyping are indicated. B) PCR analysis of *mpodd(-)* and *mpodd::mCh* parasites showing positive integration at the 5' (5'INT) and 3' (3'INT) end as well as the presence of the selection marker (SM). Tagging of *mpodd* results in increased sizes of PCR products for 5' integration (5'INT) and the complete locus (full). Control PCRs with wild-type parasites showed no products for primers binding within the construct. The amplification of the whole locus (full) in WT parasites results in a smaller band compared to *mpodd(-)* and *mpodd::mCh* indicating that no integration took place.

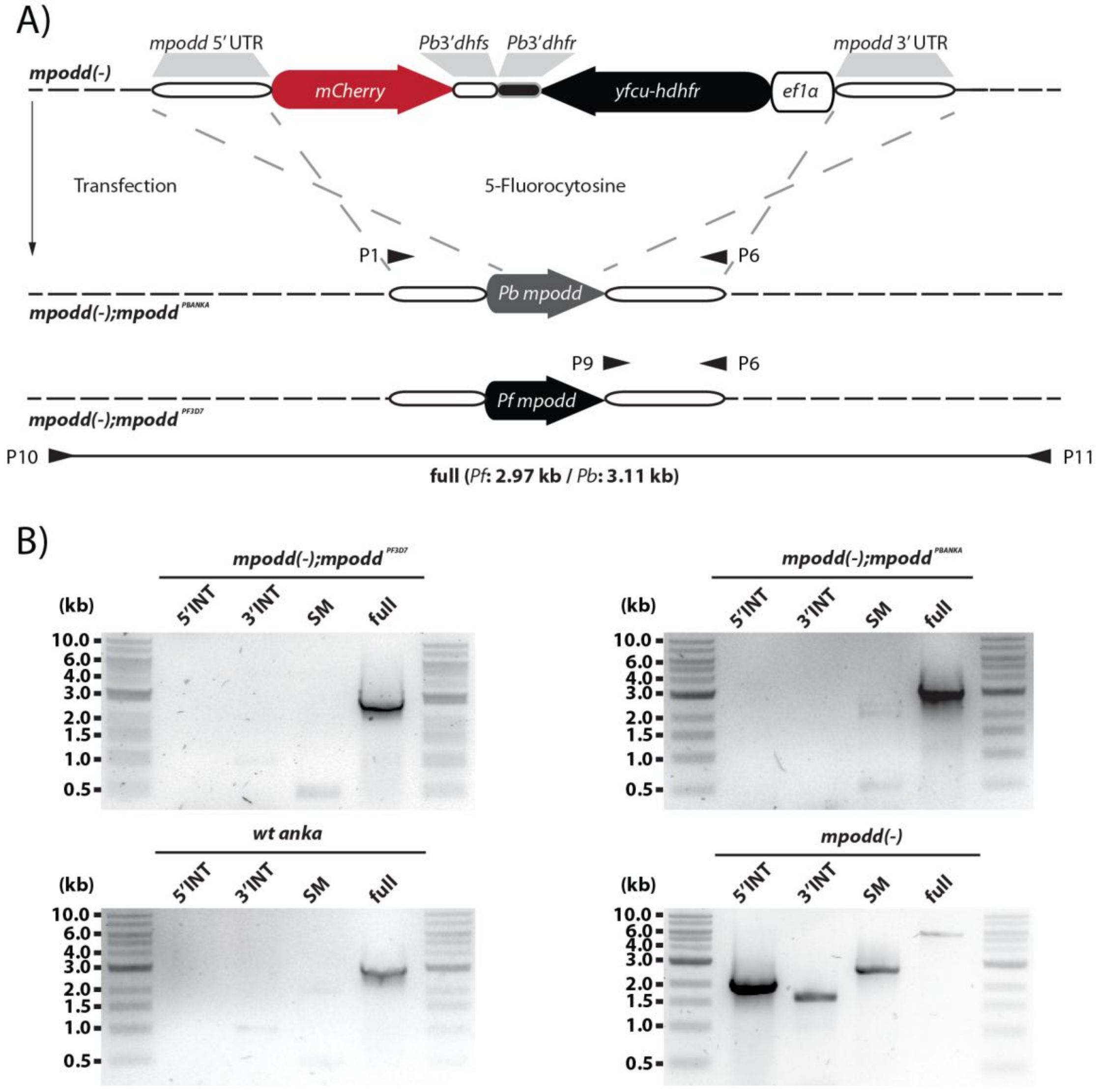

Figure S2. Generation and PCR analysis of the complemented parasite lines *mpodd(-);mpodd^{PBANKA}* and *mpodd(-);mpodd^{PF3D7}*

Schematic representation of complementation strategy A) *mpodd(-)* parasites were complemented with *Pb mpodd* as well as the homologue *Pf mpodd* from *P. falciparum 3D7*. Knockout parasites were either transfected with DNA containing the *Pb mpodd* gene or the intron-less *Pf mpodd* gene. ORFs were flanked by 1kb sequences downstream and upstream of the native locus on chromosome 12 from *P. berghei* for double crossover homologous recombination. *mpodd(-);mpodd*^{PBANKA}, and *mpodd(-);mpodd*^{PF3D7} parasites were selected with 5-fluorocytosine and cloned. Location of primers and length of PCR fragments used for genotyping are indicated. B) All complementations as well as WT show no PCR products with primers which bind within mCherry or the positive-negative selection cassette *hdhfr-yfcu* (5'INT, 3'INT and SM). PCR products for the complete locus (full) of complemented parasite lines show no difference to WT indicating that the native conditions are restored. Sequencing of the complete locus (full) of *mpodd(-);mpodd*^{PBANKA} and *mpodd(-);mpodd*^{PF3D7} parasites showed correct complementation.

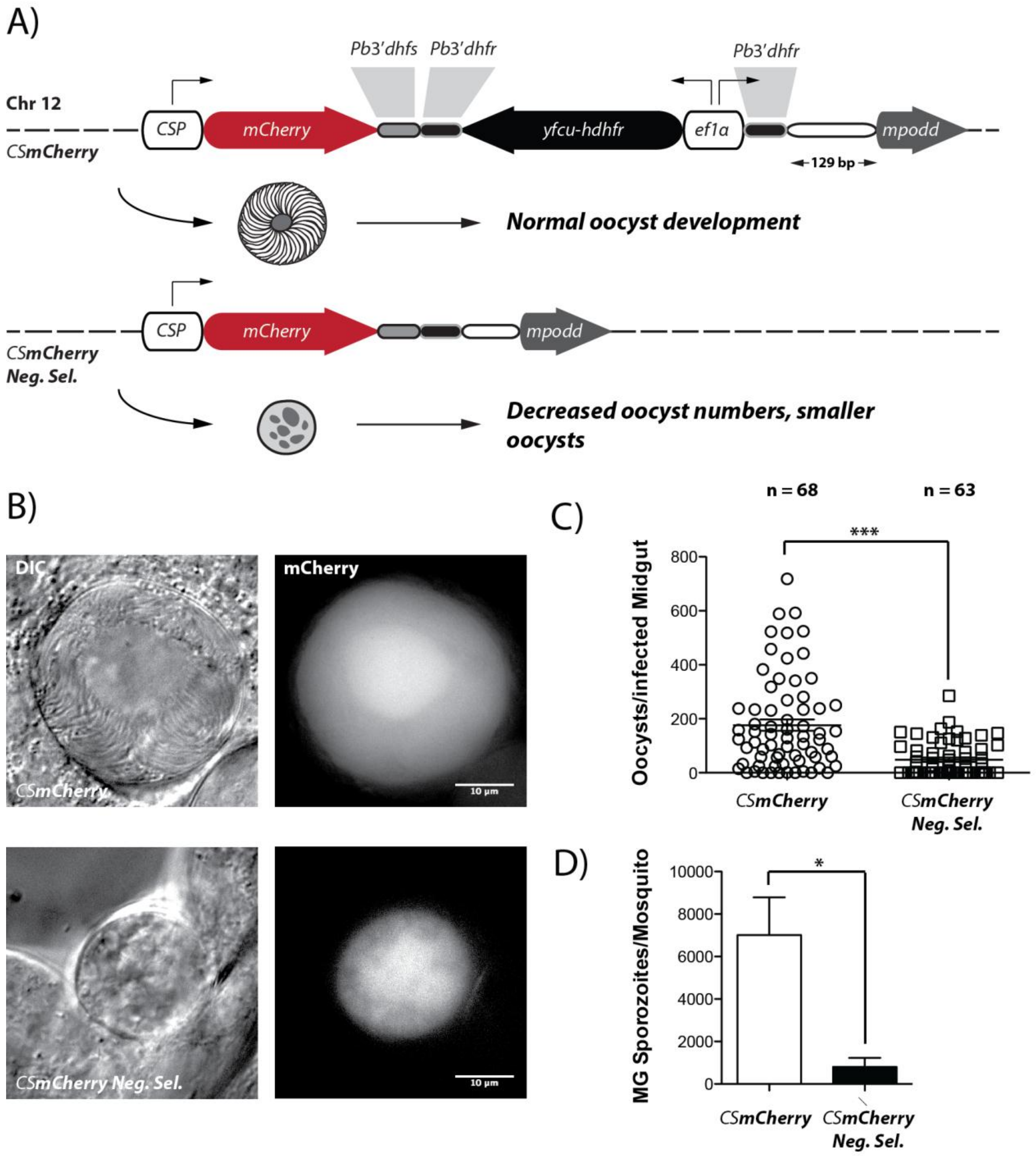

Figure S3. Integration on chromosome 12 can affect malaria transmission

Schematic representation of the gene integration strategy in chromosome 12 prior and after negative selection. A) Shown are transgenic lines expressing the fluorescence marker mCherry under regulation of the circumsporozoite protein (*CSP*) promoter. The directionality of promoter activity is indicated by arrows (*CSP and ef1α*). Note that *ef1α* shows bi-directional promoter activity. B) Images of representative oocysts 12 days post infection. Scale bar: 10 µm. C) and D) Numbers of oocysts and midgut sporozoites 11-13 days post

infection. Shown is the mean and standard error of three infection experiments. ***p<0.001 (Mann-Whitney two-tailed test), *p<0.05 (Mann-Whitney one-tailed test).

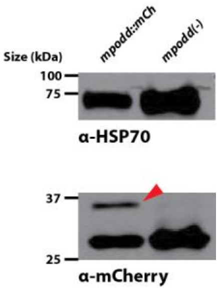

Figure S4. Expression of mCherry tagged MPODD in schizont cultures of *mpodd::mCh* and *mpodd(-)*

Western blot of *mpodd::mCh* and *mpodd(-)* schizont cultures probed with anti-mCherry and anti-HSP70 antibodies. The lower band represents mCherry (26 kDa) while the upper band shows the loading control HSP70 (75 kDa). The band representing the fusion protein MPODD::mCherry (37 kDa) is marked with a red arrowhead.

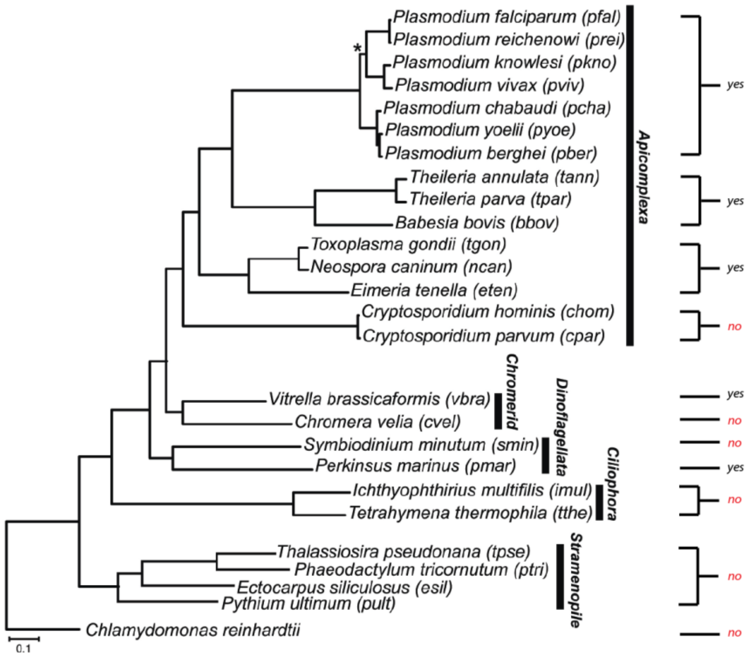

Figure S5. Presence of MPODD homologues in Apicomplexa, chromerids and dinoflagellates

The presence/absence of an MPODD homologue in a species is indicated on the right side of the phylogenetic tree (modified from [53]). The most ancient homologue was identified in the dinoflagellate *Perkinsus marinus* while no homologues could be identified in *Chromera velia*, *Symbodinium minutum* and *Cryptosporidium* species.

Table S1. Primer sequences used.

Primer sequences used for the generation of the different parasite lines, genotyping and performance of the reverse transcriptase (RT)-PCR.

Table S2. Summary of quantification: *in vivo* experiments

Data of *in vivo* experiments to determine blood stage growth rates and prepatencies of sporozoite infections of all generated parasite lines. Prepatencies are shown as the mean of all infected mice. N/D: not determined since mosquitoes were not observed to have sporozoites.

Line	Asexual blood stage growth rate (fold/24h)	Bite back prepatency (d) (n=4)	10000 sporozoites i.v. injection prepatency (d) (n=4)
wt anka	10.2 ± 1.1 (n=5)	3.25	3
mpodd(-)	9.0 ± 0.3 (n=3)	N/D	N/D
P. berghei mpodd complementation	8.8 (n=1)	3.75	3
P. falciparum mpodd complementation	10.3 ± 1.0 (n=3)	3.25	3.25

Table S3. Bioinformatic details of all MPODD homologues used in this study.

Details about MPODD homologues used for complementation and sequence comparisons. The following databases were queried: plasmodb v 26 (www.plasmodb.org); eupathdb v 26 (www.eupathdb.org); genbank (www.ncbi.nlm.nih.gov).

Species	GeneID/EstID/Location	search algorithm employed			
		blastP	tblastN	tblastN	RT-PCR
		protein	EST	genome	
Plasmodium berghei ANKA	PBANKA_1222220	plasmodb			RT-PCR
Plasmodium chabaudi chabaudi	PCHAS_1222850	plasmodb			
Plasmodium yoelii yoelii 17X	PY17X_1225400	plasmodb			
Plasmodium yoelii yoelii YM	PYYM_1224900	plasmodb			
Plasmodium falciparum 3D7	Pf3D7_08_v3(425171-424797)			plasmodb	RT-PCR
Plasmodium vivax Sal-1	Pv_Sal1_chr01(439744-440244)			plasmodb	
Plasmodium knowlesi strain H	PKNH_01_v2(484744-485255)			plasmodb	
Vitrella brassicaformis CCMP3155	Vbra_1532	eupathdb			
Sarcocystis_neurona_SN3	SN3_01100145	eupathdb			
Toxoplasma_gondii_GT1	TGGT1_223485	eupathdb			RT-PCR
Toxoplasma_gondii_VEG	TGVEG_223485	eupathdb			
Hammondia_hammondi_strain_H.H.34	HHA_223485	eupathdb			
Sarcocystis_neurona_SN1	SRCN_5331	eupathdb			
Cytauxzoon_felis_strain_Winnie	CF002938	eupathdb			
Theileria_parva_strain_Muguga	TP02_0426	eupathdb			
Babesia_bigemina_strain_BOND	BBBOND_0400230	eupathdb			
Theileria_orientalis_strain_Shintoku	TOT_030000548	eupathdb			
Eimeria_falciformis_Bayer_Haberkorn_1970	EfaB_PLUS_24117.g1976	eupathdb			
Theileria_equi_strain_WA	BEWA_015050	eupathdb			
Babesia_bovis_T2Bo	BBOV_IV007840	eupathdb			
Babesia_microti_strain_RI	BBM_III02825	eupathdb			
Theileria annulata	tann.chr03(622621...622962)			genedb	
Neospora caninum	BN1204_048985	genbank			
Eimeria tenella	CD346117.1		genbank		
Perkinsus marinus	PMAR011745	genbank			
Oxyrrhis marina	EG742930.1		genbank		
Karlodinium veneficum	EC156715.1		genbank		
Alexandrium tamarense	CK783256.1		genbank		

Document S1. gDNA and cDNA sequences of *Pb mpodd*, *Pf mpodd* and *Tg mpodd.*

Sequencing results of cDNA and gDNA from mixed blood stages and cultured *T. gondii*.

Plasmodium berghei ANKA

Pb mpodd, genomic sequence

ATGTTTAAATTACCATTTTATAGCTTTAACAAAAATTCATTGTTGGTTCAATGCTGTAAAGGAGCAGTTGCTTAT
TATATTCCGCAAAACTTAGTAGTTATTAGCATTTTTTTTTATTACCAATATAATgtaaagcacaaaaatattaaa
aattaagaaaaaaataaattattacacacaattgtagatatatctttataataaaaattgtttttttatttcttt
ttttcattttaattattttatagAAAAGCCAACTAACAAGTGGAAAGAGGGTGCGAATACAAAACAGTGATGAAT
CAATAAATAATTTTTTAAAAGGAAAACAGTTAACAAAGGACAACATCGAAATACAAAAAAAACGAAACATATATA
CCGTATCCCTGATATAA

Pb mpodd, open reading frame, mRNA

ATGTTTAAATTACCATTTTATAGCTTTAACAAAAATTCATTGTTGGTTCAATGCTGTAAAGGAGCAGTTGCTTAT
TATATTCCGCAAAACTTAGTAGTTATTAGCATTTTTTTTTATTACCAATATAATAAAAGCCAACTAACAAGTGGA
AAGAGGGTGCGAATACAAAACAGTGATGAATCAATAAATAATTTTTTAAAAGGAAAACAGTTAACAAAGGACAAC
ATCGAAATACAAAAAAAACGAAACATATATACCGTATCCCTGATATAA

Plasmodium falciparum 3D7

Pf3D7 mpodd, genomic sequence

ATGATTAAATTACCTTTTTATAGTTTTAATAAAAATTCCTTACTTGTACAATGTTTGAAATCAGCATGTGTATAT
TACATCCCTCAGAATTTATTGGTTCTTAGTGCTTTTATTTTTTATAAATATAAGgtaatatatacacatatatat
atatatgtgtgtgtatgtttttttttttttttgtttatttttgaatgctcttatgttttaaaataatattttta
tttgtttagAAAAGTCAATTTACTAATGGCAAAAGGATTCCTATAACAAACTGCAATGAAGCAATACATAATTTT
GTGGTTGAAAAAAAATTAAAAAAAGATGACATACAAATACAGAAAAAAAAAAAATGTATACATCATATCGTTAGTT
TGA

Pf3D7 mpodd, open reading frame, mRNA

ATGATTAAATTACCTTTTTATAGTTTTAATAAAAATTCCTTACTTGTACAATGTTTGAAATCAGCATGTGTATAT
TACATCCCTCAGAATTTATTGGTTCTTAGTGCTTTTATTTTTTATAAATATAAGAAAAGTCAATTTACTAATGGC
AAAAGGATTCCTATAACAAACTGCAATGAAGCAATACATAATTTTGTGGTTGAAAAAAAATTAAAAAAAGATGAC
ATACAAATACAGAAAAAAAAAAAATGTATACATCATATCGTTAGTTTGA

Pf3D7 mpodd, synthesized sequence (GeneArt)

ATGATTAAATTACCTTTTTATAGTTTTAATAAAAATTCCTTACTTGTACAATGTTTGAAATCAGCATGTGTATAT
TACATCCCTCAGAATTTATTGGTTCTTAGTGCTTTTATTTTTTATAAATATAAAAGTCAATTTACTAATGGCAAA
AGGATTCCTATAACAAACTGCAATGAAGCAATACATAATTTTGTGGTTGAAAAAAAATTAAAAAAAGATGACATA
CAAATACAGAAAAAAAAAAAATGTATACATCATATCGTTAGTTTGA

***Toxoplasma gondii* GT1**

Tg mpodd, genomic sequence

ATGAGAGTTCCTGGATTTCCTTCCCTCACTTTCCGAGCAGACTCGTTACTCGTTCAGTGCTGCATCGCCGCATGC
GTCTGgtaagccaaccgcgttcatgttttgccttcctttcagccggagctgtctttgcgggggcgcaccttgaca
agaacacggagctggcaatcgtgcagatggtcatgcttctgcgagagtgtgcggctggaatctcgcacgcagttc
tgtgtttctctttcgactgtccctgtagcgcaactctcttcacggtgccgtagaaaggaagccccgaatttgcat
ccatctggaagcgaaaaccgcgccgggaaacgaaattcggaatacctaatgggcaggcacgcagtcgacagagcc
gagaggcttgatacctttcgttgtagtctttgaacggaccgctctctcgattgatttcgctgccgcaaaccgga
cgtaaagaactccagaattctttgccgagtctcgccgttcggaggcggcactccttgaatgcgaacttccttctg
cgtggttttcggctgacttacgaggctctcaaaggtccgagtttttgtgcatatccaagctttcataatgattg
gaaatctagtgcgggcgcttccccagtctgttgcaaagcattcactgtgttccccgtatactctgcaagtagttc

ggtgtataagctgatcatcgtgactcctagagccactgaatgcccacaagcgccgaacattctatcagccttcca
tgatgtggtgatatcctaatgcagGTATGGTCCTCAAGATGCAGCGGCCGTTGGGCTCATGTACTGGCTGGGACG
TCAGgtacgtgcaggagattcatgatttttcctttctgcaggactcgttccccttggcaccaaatcgcccat
gttttgtatacatttgcatgtctgcagccacacaccatcatatatgaagaaacacgaacaaatgtagatacgtat
atagagatattcagagatatttgtgtctgatgtagacgcccgtgctggtcgcaggcaactatgttccagggaaaa
agctgtgcctttcaggcttgtcaaagtcttgtcacgcgagcttggtgtgtcgtaccaacgctagtgtatgcatgt
gagagaccgtcgtagcggcattcgcctatatatatatatatatatatatatatatatatatatatttattcat
gcagagagagacttgtactcacactttctggctgaaaagagaggtaaccccagtcgctgtccagatgcaagcaag
tatttattcaaggatctcgctctgatacacaagacgcgtccctcacagctcggccacttacctatacgggcatca
tggtattcgtatgcggcgctgcgacttcaccgttgtcatccggggcgcccgtagcgctaacagggggggacgag
aggtttcctcgaacttttgttcgtgcctttgtttcattagagtttgttccagtctgcagttagacaagtcgaact
tcatgtgcgtacaaaagtacgcgtgcaggggtgtctggggaacacagagaatttccggtgtgtctgtttagtacg
cacttacacatgcgtgcgtcgaacaggaagagatatggaaatgcagatgttggaaacgagattggtgagcaatat
gttgtggtcacagCACGGCAAGAAAGTGACGCCTCCTCACAAGCTCGCTTCTGACGGCCAGACAGCCCTCCAGGA
CTTCCTTAGACGCAAGAACCTTACTAGCCAGCAGGTCACAGTGGACTGGAAAGGCGCCAACGGCTACGTGAATCT
CCGAAGCTGA

Tg mpodd, open reading frame, mRNA

ATGAGAGTTCCTGGATTTCCTTCCCTCACTTTCCGAGCAGACTCGTTACTCGTTCAGTGC
TGCATCGCCGCATGCGTCTGGTATGGTCCTCAAGATGCAGCGGCCGTTGGGCTCATGTAC
TGGCTGGGACGTCAGCACGGCAAGAAAGTGACGCCTCCTCACAAGCTCGCTTCTGACGGC
CAGACAGCCCTCCAGGACTTCCTTAGACGCAAGAACCTTACTAGCCAGCAGGTCACAGTG
GACTGGAAAGGCGCCAACGGCTACGTGAATCTCCGAAGCTGA

Introns are displayed in small letters and highlighted in grey.

Exons are displayed in capitals.
